# The diagnostic quandary of magnetic resonance imaging-negative Hirayama disease: a case report

**DOI:** 10.1186/s13256-020-02453-2

**Published:** 2020-08-21

**Authors:** Rajeev Ojha, Sumit Shahi, Gaurav Nepal, Arjana Shakya, Bikram Prasad Gajurel, Ragesh Karn, Reema Rajbhandari, Niraj Gautam

**Affiliations:** 1 Department of Neurology, Tribhuvan University Teaching Hospital, Maharajgunj, Kathmandu, 44600 Nepal; 2 Department of Medicine, Tribhuvan University Chitwan Medical College, Bharatpur, Chitwan 44200 Nepal; 3 Maharajgunj Medical Campus, Tribhuvan University Institute of Medicine, Maharajgunj, Kathmandu, 44600 Nepal; 4 BP Koirala Institute of Health Sciences, Dharan, 56700 Nepal

**Keywords:** Hirayama disease, monomelic amyotrophy, rare neurological disorder, anterior horn cell disease

## Abstract

**Background:**

Magnetic resonance imaging (MRI) features are typical findings in Hirayama disease (HD) and are useful diagnostic entities but may not be present in all patients.

**Case presentation:**

We present the case of a 22-year-old Nepalese man who presented with insidious onset of weakness of his right upper limb of more than 5 years duration. His weakness was progressive for the first 3 years, and then remained static. On examination, weakness of the interossei, thenar, hypothenar, flexor, and extensor muscles were present in his right upper limb, power was normal in his left upper and bilateral lower limbs. Minipolymyoclonus was present in both upper limbs, less prominent on the left side. Electrophysiological findings showed motor axonal neuropathy in his right upper limb, neurogenic discharges and fibrillations, and fasciculations in both upper limbs. Contrast magnetic resonance imaging (MRI) of his cervical spine in flexion was normal. Our patient was diagnosed with HD based on clinical and electrophysiological findings. Our patient was advised to use a cervical collar and regular physiotherapy and was found to have subjective benefit.

**Conclusion:**

A normal cervical MRI does not rule out HD and the diagnosis can also be made based on clinical and electrophysiological studies. Progressive distal upper limb weakness or tremor in young patients should be evaluated for HD, because early diagnosis and intervention might halt the progression.

## Background

Hirayama disease (HD), or monomelic amyotrophy, is a rare neurological condition affecting young Asian men. It is a sporadic juvenile muscular atrophy of the distal upper extremities predominantly affecting the C7, C8, and T1 myotomes. The typical clinical features include insidious onset and slow progression of unilateral or asymmetrically bilateral atrophy of the forearm and hand muscles with sparing of the brachioradialis muscle, giving an appearance of oblique amyotrophy [1, 2]. HD is also an anterior horn cell disease, however, unlike amyotrophic lateral sclerosis (ALS), HD only progresses for about 5 years and then reaches a plateau with subsequent stabilization [1, 3].

The pathophysiology of HD is poorly understood. Due to the uneven growth of the vertebral column and the dura mater, the dura mater becomes shorter and stretched. These changes can shift the posterior dural sac anteriorly when flexing the neck, causing the compression of the cervical spinal cord against the vertebral body. During this process, the anterior vertebral plexus is compressed with resultant congestion of the posterior venous plexus. This results in microcirculation changes and focal ischemic changes in the anterior horn cells of the lower cervical cord [2, 4].

The disease is more common in Japan and other Asian countries, but cases have been reported in other parts of the world. The diagnosis of HD is based on clinical findings, nerve conduction tests (NCT), electromyography (EMG), and magnetic resonance imaging (MRI) of the cervical spine in neutral and flexed positions [1, 2]. However, not all cases of HD have typical MRI findings, which can cause diagnostic difficulties and lead to misdiagnosis. We introduce here a rare entity, an MRI-negative case of HD from Nepal.

## Case presentation

**Fig. 1:**
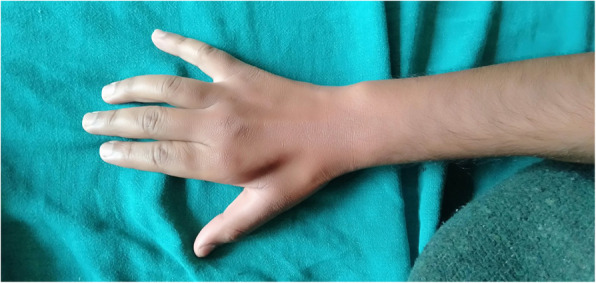
Severe wasting of first interosseus muscle with mild atrophy of rest of the interossei muscles

**Fig. 2:**
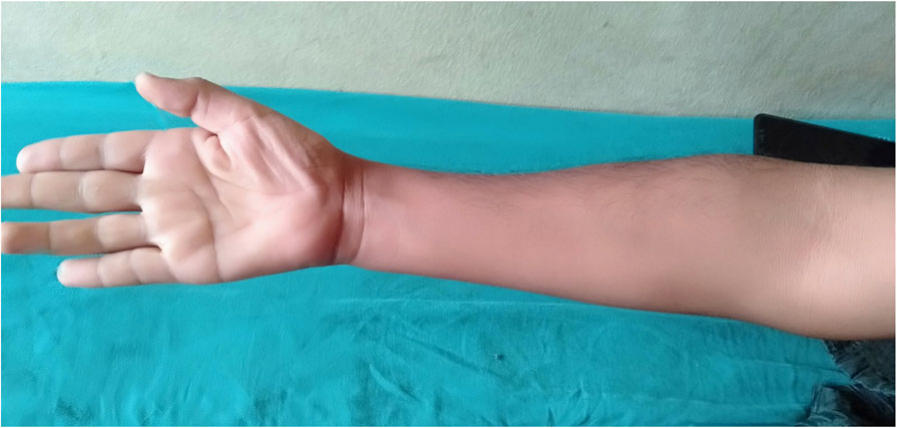
Wasting of forearm muscles, thenar muscles with relative sparing of brachioradialis muscle

A 22-year-old Nepalese man, who did not smoke or use alcohol, presented as an outpatient to Neurology at Tribhuvan University Teaching Hospital with insidious onset of weakness of his right upper limb of more than 5 years’ duration. He initially noticed difficulty in sustaining his grip while holding objects in his hand, which slowly progressed to difficulty with his fine motor skills such as buttoning or unbuttoning, writing, combing his hair, and holding a spoon. He also noticed a tremor and a gradual reduction in the muscle bulk of his right hand. After 2 years of symptoms in his right hand, he had tremors in his left hand as well, but without any weakness. His symptoms were progressive for the first 3 years and static since then. There was no history of neck pain, numbness, tingling, or burning sensation. Our patient denied any loss of sensation, autonomic dysfunction, and changes in bowel or bladder habit. He did not give any history of any form of trauma, surgery, medicine intake, vaccination, and exposure to toxins/heavy metals. Our patient is now a bachelor-level student with a part-time occupation for the last 2 years as a salesman in a department store, and there is no family history of similar complaints or neurological disease. On admission to our hospital, his general condition was fair and his vital signs were stable: blood pressure of 100/70 mm Hg; pulse of 68/minute; temperature of 98 °F and his respiratory rate was 16/minute. On general examination, he was alert and well-oriented to time, place, and person. There was no jaundice, clubbing, cyanosis, lymphadenopathy, dehydration, pallor, and edema present. His spine had no abnormal curvatures, no tenderness or restricted movement. Bilateral chest findings were normal, his heart sounds were normal without murmur, and his abdomen was non-tender without organomegaly. On neurological examination, his higher mental functions, cranial nerves, coordination, sensations, and gait were normal. A neurological examination showed weakness and atrophy of his right hand with no involvement of muscles of the proximal arm. Atrophy was prominent in the first web space in the dorsum of his right hand (Fig. 1). Muscle power was assessed by the Medical Research Council scale where weakness was observed on the right side: interossi (2/5), elbow flexon and extension (4/5), wrist flexon and extension (3/5), thenar and hypothenar were atrophied, but the brachioradialis was spared (Fig. 2). The power of his left hand and forearm was normal. Tone was reduced around his right wrist and elbow. Minipolymyoclonus was present in the fingers of his bilateral upper limbs, less prominent on the left side. His deep tendon reflexes were bilaterally symmetrical and normal in his upper and lower limbs except the brisk reflex was noted in the right supinator. Sensation was intact and bilaterally symmetrical in his upper and lower limbs. The plantar reflex was normal; a bilaterally downward response and the Hoffman sign were absent in both upper limbs.

A complete blood count (hemoglobin, total leukocyte count, differential leukocyte count, and platelet count), blood urea, serum creatinine, serum electrolytes, liver function tests, thyroid function tests, erythrocyte sedimentation rate, C-reactive protein, creatine phosphokinase, viral serology markers (human immunodeficiency virus, hepatitis B and C virus, venereal disease research laboratory test), and a plain cervical spine X-ray were normal. Anti-nuclear antibodies, anti-double-stranded deoxyribonucleic acid (DNA) antibodies, and anti-neutrophil cytoplasmic antibodies were absent.

**Fig. 3:**
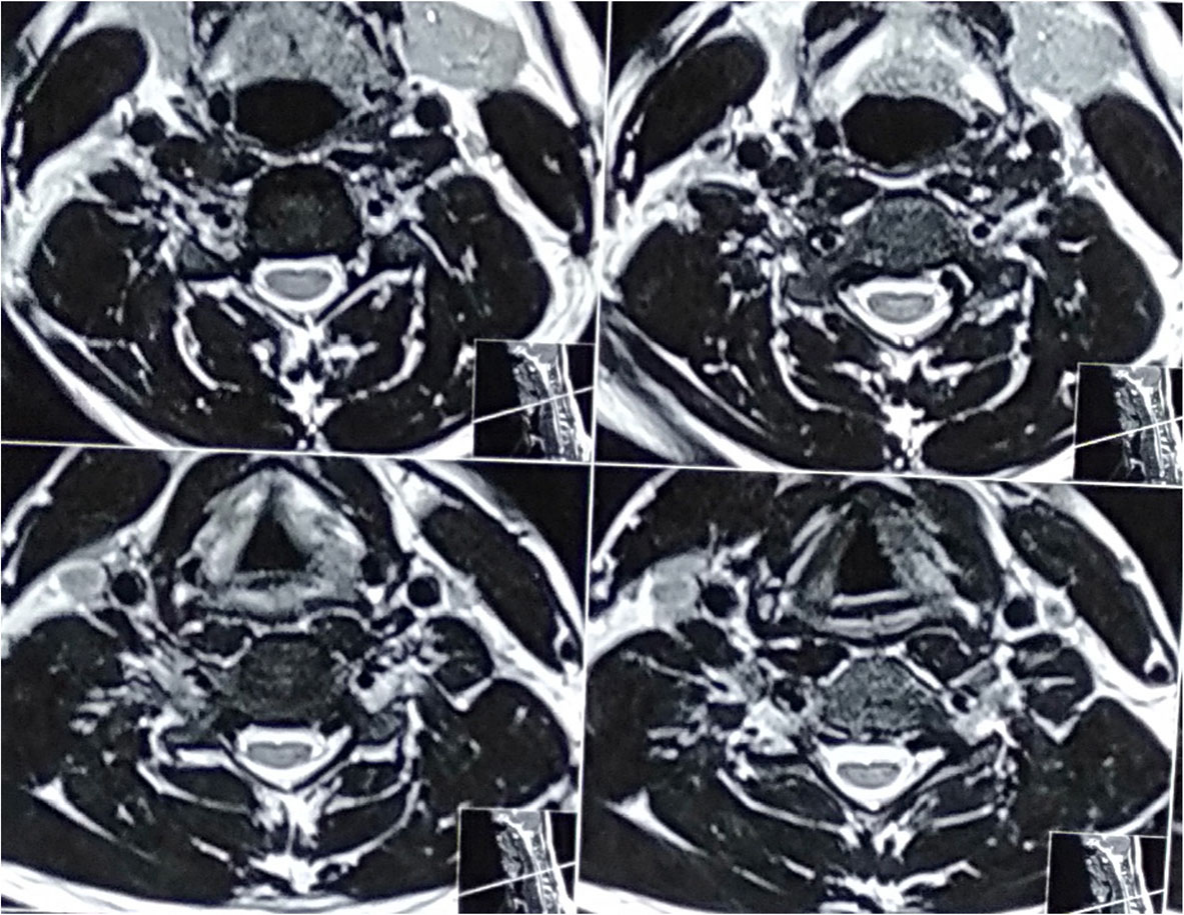
Axial T2-weighted cervical images without prominent cord hyperintensity or cord displacement

**Fig. 4:**
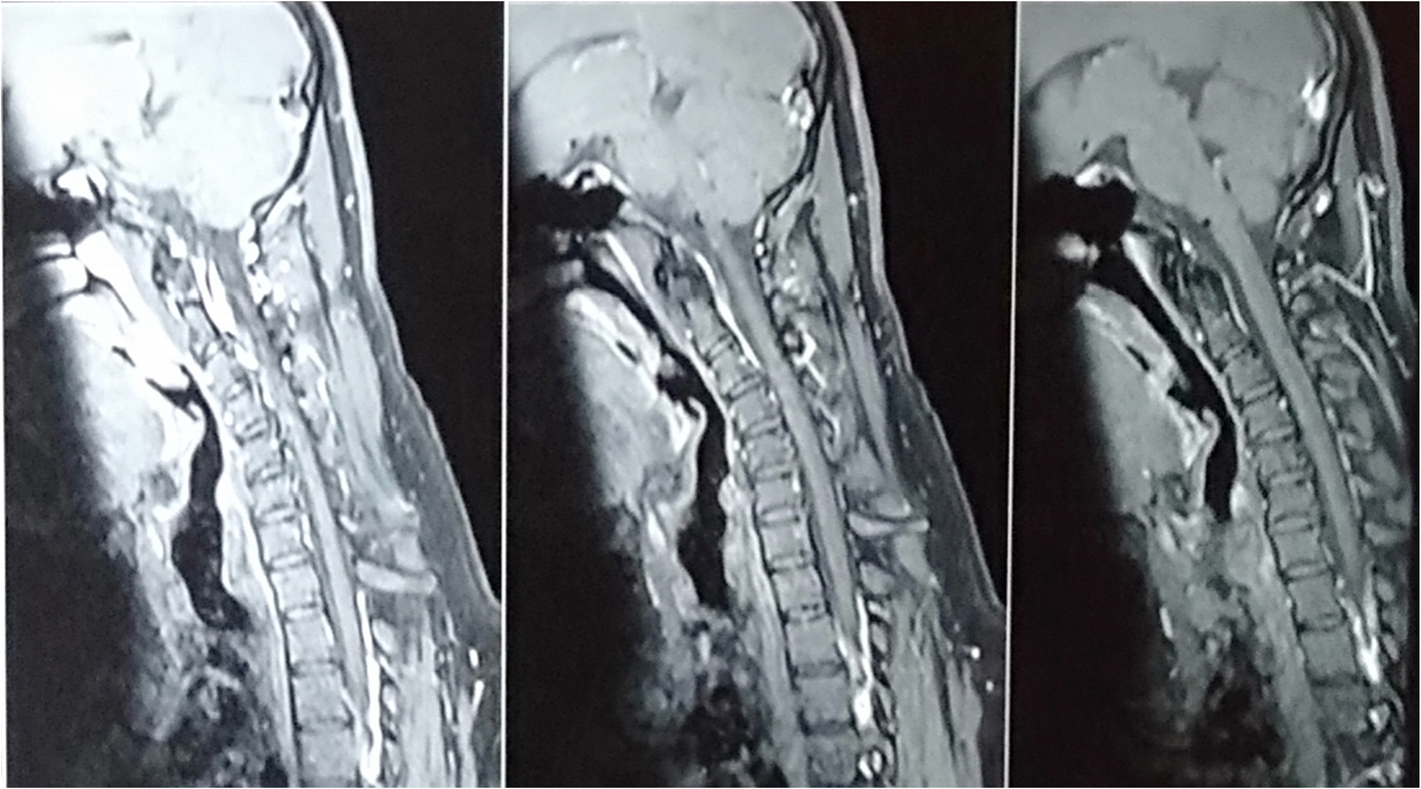
Sagittal T1-weighted contrast with cervical flexion images without epidural enhancement or cord displacement

NCT showed features of motor axonal neuropathy with reduced compound motor action potential in the right median and ulnar nerve; however, it was normal in range on the left side. A neurogenic pattern of discharges along with fibrillation and fasciculations was noted in a needle EMG in the bilateral first dorsal interossei, and right biceps and triceps. A recent cervical MRI flexion with contrast (Figs. 3 and 4) showed no abnormality in the visualized spinal cord, dural space, or cord displacement. A previous MRI had been done 2 years ago, which was also normal.

Hirayama disease was diagnosed on the basis of clinical history, examination, NCT, and EMG studies, but the characteristic findings were absent on the MRI (1.5 Tesla). Our patient was advised to use a cervical collar along with cervical extension and distal upper limb physiotherapy and asked to follow up at regular intervals. Because of geographic and financial constraints, our patient could not come for follow-up. However, at the 3-month telephone follow-up, our patient felt that his muscle strength was better than before, and there was improvement in his fine motor activities.

## Discussion and conclusions

Our patient presented with young onset of progressive unilateral distal hand weakness with atrophy and tremulousness of both hands of 5 years’ duration, which was static for 2 years without any bulbar, sensory, or lower limbs symptoms. A cervical spine MRI with contrast during flexion was found to be normal. Absence of positive findings on MRI is uncommon, which might be a diagnostic quandary to neurologists and sometimes HD can be misdiagnosed as ALS, spinal muscular atrophy, and post-polio syndrome. A pattern of weakness and progression of disease along with proper electrophysiological interpretation will help in confirming the diagnosis of HD.

HD is named after Keizo Hirayama, who first described the disease in 1959 [1]. HD is more common in Asian men, aged between the second and third decades of life. It is characterized by insidious onset and slow progression of unilateral or asymmetrically bilateral atrophy of the forearm and hand muscles with sparing of the brachioradialis muscle. There is no pain, altered sensations, and autonomic changes associated with muscle atrophy. The progression is slow, usually over 5 years, before reaching a plateau and stabilization [1, 2, 5]. Even after the first mention more than 60 years ago, the exact etiology, diagnostic criteria, and treatment of HD are still lacking. As mentioned in the introduction, the widely accepted theory is the advancement of the posterior cervical dura mater, which results in compression of the cervical spinal cord against the posterior margin of the vertebral body, subsequent compression of the venous plexus, microcirculation disturbance, and anterior horn cell ischemia.

As a norm, the most widely accepted case definitions for HD include: weakness and atrophy of the forearm and hand muscles, unilateral or asymmetric bilateral involvement, young, insidious onset with gradual progression followed by stabilization, no lower limb involvement, no sensory or autonomic disorders, and distinctive NCT, EMG, and MRI findings. Our patient has a similar history of clinical manifestations, patterns of atrophy and weakness, progressiveness, and finally was in a static state for more than 2 years. The differential diagnosis of HD includes distal forms of spinal muscular atrophy, ALS, post-poliomyelitis syndrome, traumatic myelopathy, toxic neuropathy, spinal tumors, syringomyelia, myelopathy associated with cervical spondylosis, and anterior interosseous or deep ulnar neuropathy. With medical history, examination, and NCT/EMG features, spinal muscular atrophy, ALS, post-polio syndrome, multifocal motor neuropathy, anterior interosseous or deep ulnar neuropathy, and toxic neuropathy were ruled out. Cervical MRI helped to rule out syringomyelia, traumatic myelopathy, spinal tumors, and other spinal diseases.

In HD, the EMG shows neurogenic patterns, fibrillations, and fasciculations. NCT shows decrease in the compound muscle action potential of the median and ulnar nerves in the affected limb [2]. Cervical MRI performed in the neutral and flexed positions significantly contributes to the diagnosis of HD. Characteristic findings include local cord atrophy, abnormal cervical spine curvature, and asymmetric cord flattening in the neutral position [6]. Loss of dural attachment, dorsal dural advancement, enlarged epidural space with flow void, and increase in laminodural space distance are the findings in the flexed position [6, 7]. The proposed MRI protocol includes routine cervical spine sequences taken at a neutral position and with cervical spine flexion at 20° to 40° [4, 6]. Even when MRI is performed with flexion, an insufficient flexion angle can obscure the findings and prevent timely diagnosis. If the flexion MRI is normal and if the index of suspicion is high, a serial flexion MRI of the cervical scan can be performed. However, in some cases, including ours, there were characteristic clinical features with a progression pattern, NCT/EMG findings, and negative MRI results despite serial MRI in the proper flexion angle [2, 8]. Symptoms in patients with HD are due to the result of pathological changes in the anterior horns of the lower cervical cord, such as ischemia and necrosis. In our patient, the severity of symptoms was moderate, and he showed some improvement with minor intervention like the use of a cervical collar and physiotherapy. Cord pathology might be minimal in such a patient as to visualize significant cord atrophy or myelopathic changes on MRI. Even in cervical flexion contrast imaging, no epidural mass or anterior shift of the dural canal was seen, suggesting some other cervical posture might cause congestion of the posterior internal venous plexus. However, limitations such as technical expertise and the timing of contrast and image recordings could be possible.

Although HD has been emphasized as a self-limiting and non-progressive disease, it can cause social disability in those with a complete loss of hand function. In the worst case, after a period of stabilization, it may develop into severe spastic paraparesis [2]. Therefore, accurate diagnosis and regular periodic follow-up are essential. Early intervention can minimize disease progression. Conservative treatments, such as the use of cervical braces to prevent cervical spine flexion, have been shown to reverse disease progression [4, 8, 9]. Surgical treatment is required only in selective cases, where progressive deterioration occurs despite conservative treatment and includes anterior cervical decompression surgery or duroplasty or reconstructions with tendon transfers [4].

HD should be suspected in young Asian men who present with insidious onset of distal upper extremities atrophy and weakness. Early diagnosis is essential to prevent social disability and unforeseen complications. Diagnosis should be done based on clinical finding, NCT, EMG, and MRI of the cervical spine in the neutral and flexion position. If flexion MRI shows no lesion, it is judicious to do serial flexion MRI. Sometimes, despite serial flexion MRI, no abnormality can be seen. Normal findings in cervical MRI does not rule out HD and the diagnosis can also be made based on clinical and electrophysiological studies.

## Data Availability

The data used in the case report are available on reasonable request.

## Abbreviations

ALS: Amyotrophic lateral sclerosis
EMG: Electromyogram
HD: Hirayama disease
MRI: Magnetic resonance imaging
NCT: Nerve conduction test
